# Pipkin fractures: epidemiology and outcome

**DOI:** 10.1007/s00068-022-01951-w

**Published:** 2022-03-25

**Authors:** Anders Enocson, Olof Wolf

**Affiliations:** 1 Department of Molecular Medicine and Surgery, Karolinska Institute, Stockholm, Sweden; 2 Department of Trauma, Acute Surgery and Orthopaedics, Karolinska University Hospital, Stockholm, Sweden; 3 Section of Orthopaedics, Department of Surgical Sciences, Uppsala University, Uppsala, Sweden

**Keywords:** Pelvic fracture, Acetabular fracture, Pipkin fracture, Epidemiology, Surgical treatment

## Abstract

**Purpose:**

To describe the epidemiology of Pipkin fractures including detailed fracture classification and outcome for joint preservation and death.

**Methods:**

We extracted data on all Pipkin fractures in the Swedish Fracture Register from 2013 to 2020 in patients ≥ 18 years. The cohort was cross-matched with the Swedish Hip Arthroplasty Register to obtain data on primary or secondary treatment with arthroplasty. We analysed data on age, sex, injury mechanism, fracture classification, treatment including secondary operative treatment with arthroplasty and mortality. Primary outcome was joint preservation.

**Results:**

In total 47 Pipkin fractures with a median age of 48 years were included. 74% of the fractures were in males. The median follow-up time was 3.5 years. The most common primary treatment was internal fixation (45%), followed by primary arthroplasty (28%), and excision of fragment (15%). Three of the 34 patients with primary non arthroplasty treatment received secondary treatment with arthroplasty. Two patients died within 30 days, and no further deaths occurred up to 1 year after injury.

**Conclusion:**

Three of four fractures occurred in males and more than half of the fractures were due to high energetic injuries. Half of the patients received internal fixation (predominantly younger patients) and 28% were treated with primary arthroplasty (predominantly older patients). The revision rate was low, and after secondary treatment with arthroplasty two thirds of the patients still had a preserved joint.

## Introduction

A Pipkin fracture is a traumatic hip dislocation with a concomitant fracture of the femoral head that was first described by Birkett in 1869 [1]. It is a rare injury and the literature is sparse, mostly consisting of smaller case-series. A recent meta-analysis found a total of 274 patients in 15 reports, each consisting of 5–39 patients [2]. The injury mechanism in the literature is most often a high-energy motor vehicle accident with a posterior dislocation of the hip, and the incidence of Pipkin fracture has been reported to 5–15% of all posterior hip dislocations [3–6]. The most established classification divides the injury into four different types depending on the location of the fracture on the femoral head, and presence of a concomitant fracture of the femoral neck or the acetabulum [7]. Published studies usually focus on subtypes of Pipkin fractures, and poor results with high complication rates and reoperation rates up to 57% have been reported [2, 8–10]. The optimal treatment is controversial, and the choices include non-surgical, surgical excision of fragment, surgical internal fixation of fragment or primary arthroplasty (with/without simultaneous acetabular fixation) [2, 9, 10].

This study aimed to describe epidemiology, treatment and outcome with respect to preservation of the hip joint and mortality, in an unselected patient cohort of patients with all types of Pipkin fractures.

## Materials and methods

### Study design and setting

The National Swedish Fracture Register (SFR) was used to identify patients aged ≥ 18 years with a registered Pipkin fracture sustained in Sweden between December 1, 2013 and June 30, 2020. The SFR started in 2011 in one department, and since then a step-wise introduction of the SFR in Sweden has led to an increased coverage and completeness over the study period. At the end of 2019 the coverage of the SFR was > 90% of the 54 orthopaedic departments in Sweden [11], and full national coverage was reached in January 2021. Completeness of hip fracture registrations compared with the National Patient Register was > 80% for hip fractures for half of the active units [11], using a process described by Bergdahl et al. [12]. In the SFR, detailed data on patient, injury and fracture characteristics as well as fracture treatment is registered prospectively at each affiliated department. The energy-level (high or low) of the injury is left to the discretion of the registering surgeon to decide in the SFR. Furthermore, the SFR is linked to the national Tax Agency, from which data on patient mortality is obtained. After retrieval of the dataset from the SFR, the local hospital for each patient was contacted to gain access to individual patient records including pre- and postoperative radiographs. Preoperative computer tomography (CT) images were used to classify the Pipkin fractures as; Type 1—inferior of the fovea, Type 2—superior of the fovea, Type 3—Type 1 or 2 with concomitant femoral neck fracture or Type 4—Type 1 or 2 with concomitant acetabular fracture [7]. The classification was done by the two authors together and a consensus was reached for each case. Primary treatment was divided into non-surgical (including closed reduction), surgery with only excision of the fragment/fragments, surgery with internal fixation of the fragment/fragments using screws or bioabsorbable nails/screws, surgery with arthroplasty or other open surgical method. Closed reduction of a dislocated hip joint prior to other intervention was not recorded specifically.

To find cases who had had a secondary operation with an arthroplasty, cross-referencing was performed with the National Swedish Hip Arthroplasty Register (SHAR). The SHAR started in 1979 and collects detailed data on all primary and secondary hip arthroplasties performed in Sweden in patients with a valid Swedish personal identification number. The completeness in SHAR of primary total hip arthroplasty procedures has been > 98% over the last 10 years compared with the NPR [13].

All patients were followed until June 30, 2021, or secondary surgery with arthroplasty, or death.

### Statistical methods

With respect to the composition of the cohort, results were expressed as numbers, proportions or median (range). The Mann–Whitney *U* test was used for comparisons of variables in independent groups. All tests were two-sided. The results were considered significant at *p* < 0.05. The statistical software used was IBM SPSS Statistics, version 28 for Windows (SPSS Inc., Chicago, Illinois).

## Results

### Epidemiology and treatment

Initially, 73 patients with a preliminary Pipkin fracture were identified in the SFR. After going through the patient files and radiographs, 26 of those were found not having a true Pipkin fracture, leaving a total of 47 patients who were included in the study. The median age of the patients was 48 (18–83) years and 12 (26%) were females. The most common injury mechanism was a fall (*n* = 19, 40%) followed by a traffic related injury (*n* = 17, 36%), other injury mechanisms (*n* = 7, 15%) or unknown (*n* = 4, 8.5%). The injury level was classified as high-energy in 27 (57%), low-energy in 11 (23%) or unable to classify in 9 (19%) patients. The median follow-up time was 41 (0–90) months (approx. 3.5 years).

Classification of the Pipkin fracture type revealed that the most common fracture was Type 4 (*n* = 26, 55%) followed by Type 2 (*n* = 13, 28%), Type 1 (n = 7, 15%) and Type 3 (*n* = 1, 2.1%).

**Fig.1:**
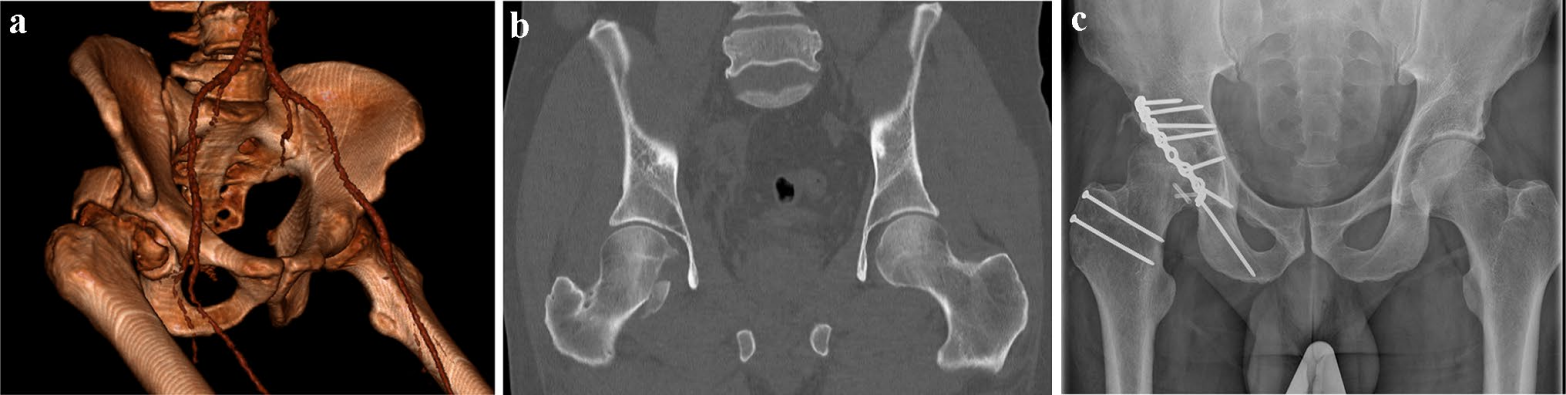
**a** 3D-CT scan before closed reduction of the hip joint in a 50 years old male after a traffic accident, displaying a Type 4 Pipkin fracture. **b** CT scan in the same patient after closed reduction of the hip joint displaying the fracture of the femoral head. **c** Postoperative x-ray in the same patient after plate fixation of the acetabular fracture and fixation of the femoral head fragment using headless screws

**Fig. 2:**
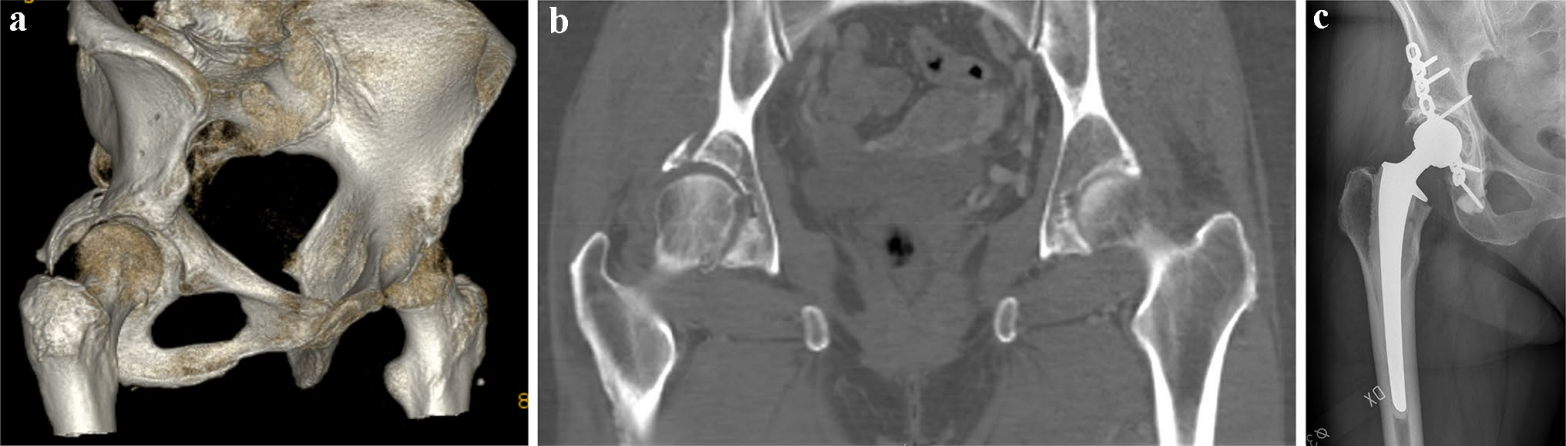
**a** 3D-CT scan in a 73 years old female after a traffic accident, displaying a Type 4 Pipkin fracture. **b** CT scan in the same patient displaying the fracture of the femoral head. **c** Postoperative x-ray in the same patient after plate fixation of the acetabular fracture and a cemented total hip arthroplasty

The primary treatment included; internal fixation (*n *= 21, 45%), arthroplasty (*n *= 13, 28%), excision (*n* = 7, 15%), non-surgical treatment (*n* = 4, 8.5%), open reduction only (*n* = 1, 2.1%) and autologous transplantation with fixation using graft from another part of the same femoral head (*n* = 1, 2.1%). All patients primary treated with arthroplasty had a total hip arthroplasty (THA) with either cemented (n = 6) or uncemented (*n* = 7) fixation (Figs. 1a–c, 2a–c).

The median time from injury to primary surgical treatment (43 patients) was 3.0 (0–14) days. There was no difference in the median time to primary surgical treatment between patients treated with an arthroplasty (*n* = 13) (3.0, 1–12 days) compared to other surgical methods (*n* = 30) (3.0, 0–14 days) (*p* = 0.8). Patients treated with primary arthroplasty were older (median 64, 51–83 years) compared to other primary surgical methods (41, 18–71 years) (*p* < 0.001). One patient treated with primary arthroplasty was further revised after 6 months due to dislocations, and 1 patient was revised after 2 weeks due to deep infection.

Detailed information on patient/injury characteristics and primary treatment in relation to fracture type is presented in Table 1.

**Table 1:** Patient and injury characteristics and treatment in relation to Pipkin fracture type

Parameter	All *n = *47	Type 1 *n = *7	Type 2 *n = *13	Type 3 *n = *1	Type 4 *n = *26
Age (years); median (range)	48 (18–83)	34 (18–55)	42 (18–76)	60 (NA)	51 (18–83)
Female gender; *n = *(%)	12 (26)	3 (43)	2 (15)	0 (0)	7 (27)
Injury mechanism, *n = *(%)					
Fall	19 (40)	2 (29)	6 (46)	1 (100)	10 (39)
Traffic related	17 (36)	3 (43)	5 (39)	0 (0)	9 (35)
Other	7 (15)	1 (14)	2 (15)	0 (0)	4 (15)
Unknown	4 (8.5)	1 (14)	0 (0)	0 (0)	3 (12)
Primary treatment, *n = *(%)					
Surgical	4 (8.5)	2 (29)	0 (0)	0 (0)	2 (7.7)
Internal fixation	21 (45)	4 (57)	8 (62)	0 (0)	9 (35)
Arthroplasty	13 (28)	0 (0)	4 (31)	1 (100)	8 (31)
Excision	7 (15)	1 (14)	0 (0)	0 (0)	6 (23)
Other	2 (4.3)	0 (0)	1 (7.7)	0 (0)	1 (3.8)

*NA* not applicable

### Outcomes

The revision rate with arthroplasty among patients not treated with a primary arthroplasty was 8.8% (*n* = 3/34). The secondary arthroplasties were performed between 7 months and 5 years and 3 months after the injury. None of these patients were further revised due to problems with the arthroplasty. Detailed information on patients operated with a secondary arthroplasty is presented in Table 2.

**Table 2:** Pipkin fracture patients operated with a secondary arthroplasty after primary treatment with other method than arthroplasty

Patient no.	Age	Gender	Fracture type	Primary treatment	Days to primary treatment	Type of secondary arthroplasty	Time from injury to secondary arthroplasty
1	64	Male	Type 4	Non-surgical	NA	Cemented THA	5 years and 3 months
2	51	Male	Type 1	Internal fixation	5	Hybride THA	7 months
3	42	Male	Type 2	Internal fixation	4	Uncemented THA	1 year and 1 month

*NA* not applicable, *THA* otal hip arthroplasty

The 30-days and 1-year mortality were both 4.3% (*n* = 2) for all patients. The 2 patients died 2 (43 years old, female, non-surgical treatment) respectively 26 (39 years old, male, surgical treatment with excision) days after the injury. Both were poly-trauma patients.

## Discussion

The primary finding in this study was a low (8.8%) rate of secondary treatment with arthroplasty in patients with primary joint preserving treatment. Secondary findings included that the typical patient was a middle-aged male, with a Type 4 fracture after a fall, and that the mortality was low.

Few studies report on outcomes in unselected patient series such as this one. Scolaro et al. found 10% secondary surgeries with arthroplasties in patients with Type 1, 2, 3 or 4 Pipkin fractures [14]. Without presenting details, the worst outcome was for Type 3 fracture patients, who all had secondary treatment with an arthroplasty. In contrast to our study, both hemi and total arthroplasties were used for these patients, unfortunately without giving further details on patient selection for each type of prosthesis used. Wang et al. reported one reoperation (a patient with a Type 4 Pipkin fracture) using a THA, in 12 patients (8.3%) with Pipkin Type 1, 2 or 4 fractures primary treated with internal fixation [10].

Poor results have been reported in Pipkin Type 4 fractures, with four out of seven (57%) patients being secondarily operated with a THA [8]. In our material, patients requiring secondary treatment with arthroplasty were heterogeneously distributed with one patient each having a Type 1, 2 or 4 Pipkin fracture. Further sub analysis on possible relations between fracture type and risk for secondary surgery with arthroplasty was therefore not possible. We rather conclude that we had a low, and probably acceptable, rate of secondary surgery given the complexity and severity of these injuries.

The typical patient in the current study was a middle-aged (48 years) male (74%). This age and gender distribution seem to be typical for patients with a Pipkin fracture when comparing with other studies [2, 6, 9, 10, 14]. However, when it comes to distribution of the different types of Pipkin fractures and injury mechanism, the results from our study differ somewhat from several other reports.

In our material, a Type 4 was the most frequent Pipkin fracture type affecting more than half (55%) of the patients, followed by a Type 2 (superior of the fovea) in 28% of the patients. This contrasts with Scolaro et al. who reported 47% Type 2 and only 15% Type 4 fractures in their series [14]. In the systematic review by Giannoudis et al. they also reported the Type 4 fractures being not so common (30%) [9].

Furthermore, we found that the most common injury mechanism was a fall (40%) followed by a traffic related injury (36%). This is in sharp contrast to Giannoudis et al. who found that the absolute majority (85%) of their patients had a traffic related injury, and only a minority (4.4%) had sustained the injury due to a fall [9]. Similar results were reported in the meta-analysis by Bettinelli et al. with motor vehicle accidents contributing to 78%, and a fall to 14% of the cases [2]. We can not explain why our, although large, series have a higher proportion of Type 4 fractures typically caused by a fall, rather than other fracture types caused by a traffic related injury. As a speculative explanation, we propose that a fall, as injury mechanism, is more likely to cause a Pipkin fracture including a concomitant acetabular fracture compared to traffic injuries. One could also speculate that our more recent study could indicate more of osteoporotic fracture patients, as these have increased over the last decade [15], although our age and gender distribution was not that different from previous studies [2, 9].

About one patient in four (28%) was treated with a primary arthroplasty. The literature on primary arthroplasty in Pipkin fracture patients is sparse, but Giannouds et al. reported that 3.9% of their patients had a primary arthroplasty [9]. In our series, these patients were older compared to patients treated with other surgical methods and they all had a Pipkin fracture Type 2, 3 or 4. As these fracture types in general are more severe, involving more of the weight-bearing surface of the hip joint compared to Type 1 fractures, it seems reasonable to perform a primary arthroplasty in older patients despite the fact that two of these patients were further revised. The use of primary arthroplasty in elderly patients with Pipkin fractures is further supported by reports on good clinical outcome following primary arthroplasty in combination with reinforcement rings in elderly patients with comminuted acetabular fractures [16, 17].

The mortality in this study was 4.3% at both 30 days and 1 year. We have not found any other study that reports on mortality in Pipkin fracture patients, making comparisons difficult to make. In a study including 4480 unselected trauma patients, Holtenius et al. found a 30-day mortality of 9.0% and a 1-year mortality of 10% for patients with a pelvic fracture [18]. Both deceased patients in our cohort were poly-trauma patients, and most probably other injuries than the Pipkin fractures were the major contributing factors for death.

### Strengths and limitations

A major strength of the current study was the data set including an unselected patient population from a national register—the SFR. With the Swedish personal identification number, accurate follow-up including mortality up to 1 year could be achieved. Also, joint preservation could be accurately followed by contacting each treating hospital in combination with cross-referencing with the SHAR that has excellent long-term completeness on arthroplasty procedures. Additionally, in comparison, this cohort was also one of the largest published series. Limitations includes lack of additional detailed data for each patient due to being a register study, and its retrospective design. Ideally, one would like to have had information on co-morbidity and functional status for each patient, collected prospectively.

In summary, our interpretation from the previous literature and the results from this study is that joint preserving surgery should be performed in younger patients whilst older patients can be successfully treated with a primary arthroplasty.

## Data Availability

Not applicable.

## Data Availability

Data can be made available on request to the authors.

## References

[1] Birkett J (2000). Description of a dislocation of the head of the femur, complicated with its fracture; with remarks by John Birkett (1815-1904). 1869. Clin Orthop Relat Res.

[2] Bettinelli G, Placella G, Moharamzadeh D, Belluati A, Salini V (2021). Articular femoral head fracture management: a meta-analysis of literature. Indian J Orthop.

[3] Droll KP, Broekhuyse H, O'Brien P (2007). Fracture of the femoral head. J Am Acad Orthop Surg.

[4] Epstein HC, Wiss DA, Cozen L (1985). Posterior fracture dislocation of the hip with fractures of the femoral head. Clin Orthop Relat Res.

[5] Hougaard K, Thomsen PB (1988). Traumatic posterior fracture-dislocation of the hip with fracture of the femoral head or neck, or both. J Bone Joint Surg Am.

[6] Sahin V, Karakas ES, Aksu S, Atlihan D, Turk CY, Halici M (2003). Traumatic dislocation and fracture-dislocation of the hip: a long-term follow-up study. J Trauma.

[7] Pipkin G (1957). Treatment of grade IV fracture-dislocation of the hip. J Bone Joint Surg Am.

[8] Engel JL, Johnsen P, Patel NK, Satpathy J, Mounasamy V (2021). Pipkin type IV femoral head fractures: a case series and review of literature. Eur J Orthop Surg Traumatol.

[9] Giannoudis PV, Kontakis G, Christoforakis Z, Akula M, Tosounidis T, Koutras C (2009). Management, complications and clinical results of femoral head fractures. Injury.

[10] Wang J, Cai L, Xie L, Chen H, Guo X, Yu K (2019). 3D printing-based Ganz approach for treatment of femoral head fractures: a prospective analysis. J Orthop Surg Res.

[11] The Swedish Fracture Register. Annual Report 2019. 2020. https://registercentrum.blob.core.windows.net/sfr/r/VGR_SFR_-rsrapport-2020-SE-DIGITAL-uppslag-B1xBpRe6q_.pdf.

[12] Bergdahl C, Nilsson F, Wennergren D, Ekholm C, Moller M (2021). Completeness in the Swedish fracture register and the Swedish national patient register: an assessment of humeral fracture registrations. Clin Epidemiol.

[13] Swedish Hip Arthroplasty Register. Annual Report 2019. 2020. https://registercentrum.blob.core.windows.net/slr/r/2019-B1xpWMUSPO.pdf.

[14] Scolaro JA, Marecek G, Firoozabadi R, Krieg JC, Routt MLC (2017). Management and radiographic outcomes of femoral head fractures. J Orthop Traumatol.

[15] Lundin N, Huttunen TT, Berg HE, Marcano A, Fellander-Tsai L, Enocson A (2021). Increasing incidence of pelvic and acetabular fractures. A nationwide study of 87,308 fractures over a 16-year period in Sweden. Injury.

[16] Enocson A, Blomfeldt R (2014). Acetabular fractures in the elderly treated with a primary Burch-Schneider reinforcement ring, autologous bone graft, and a total hip arthroplasty: a prospective study with a 4-year follow-up. J Orthop Trauma.

[17] Borg T, Hernefalk B, Hailer NP (2019). Acute total hip arthroplasty combined with internal fixation for displaced acetabular fractures in the elderly: a short-term comparison with internal fixation alone after a minimum of two years. Bone Joint J.

[18] Holtenius J, Bakhshayesh P, Enocson A (2018). The pelvic fracture—indicator of injury severity or lethal fracture?. Injury.

